# Effects of Radiative Electro-Magnetohydrodynamics Diminishing Internal Energy of Pressure-Driven Flow of Titanium Dioxide-Water Nanofluid due to Entropy Generation

**DOI:** 10.3390/e21030236

**Published:** 2019-03-01

**Authors:** Ahmed Zeeshan, Nasir Shehzad, Tehseen Abbas, Rahmat Ellahi

**Affiliations:** 1 Department of Mathematics & Statistics, FBAS, International Islamic University Islamabad, Islamabad 44000, Pakistan; 2 Department of Mathematics, University of Education Lahore, Faisalabad Campus, Faisalabad 38000, Pakistan; 3 Center for Modeling & Computer Simulation, Research Institute, King Fahd University of Petroleum & Minerals, Dhahran 31261, Saudi Arabia

**Keywords:** electric field, energy loss, titanium dioxide water nanofluid, magnetic field, Poiseuille flow

## Abstract

The internal average energy loss caused by entropy generation for steady mixed convective Poiseuille flow of a nanofluid, suspended with titanium dioxide (TiO_2_) particles in water, and passed through a wavy channel, was investigated. The models of thermal conductivity and viscosity of titanium dioxide of 21 nm size particles with a volume concentration of temperature ranging from 15 °C to 35 °C were utilized. The characteristics of the working fluid were dependent on electro-magnetohydrodynamics (EMHD) and thermal radiation. The governing equations were first modified by taking long wavelength approximations, which were then solved by a homotopy technique, whereas for numerical computation, the software package BVPh 2.0 was utilized. The results for the leading parameters, such as the electric field, the volume fraction of nanoparticles and radiation parameters for three different temperatures scenarios were examined graphically. The minimum energy loss at the center of the wavy channel due to the increase in the electric field parameter was noted. However, a rise in entropy was observed due to the change in the pressure gradient from low to high.

## 1. Introduction

In recent ages, heat transfer enhancement has gained much attention in the field of technological and industrial applications—like thermal devices—owing to the cooling rate, which highly affects the manufactured product with the desired topographies. Moreover, over the years many methodologies and techniques have been used to investigate heat exchange in fluids. An electrically conducted Poiseuille fluid flow passing through straight walls with porosity was probed by Chauhan and Rastogi [1]. The electro-magnetohydrodynamic heat transfer characteristics for an incompressible fluid by virtue of ohmic and thermal dissipations was numerically testified by Pal and Mondal [2]. Authors examined that the velocity distribution increased with the increase of the electric field parameter, while the temperature decreased with the electric field parameter. Furthermore, heat transfer can be improved by presenting nanoparticles with high heat transfer characteristics in a low volume fraction within the nanofluids. Nanofluid is a new idea of nanotechnology, which is used to enhance the property of the thermal conductivity in fluids [3]. An experimental study on titanium dioxide water-based nanofluid was done by several researchers. For example, in the study by Sajadi and Kazemi [4], in which less than a 0.25% concentration of nanoparticles in water was used. Naturally, titanium dioxide occurs as the mineral compounds anatase, rutile, and brookite. It has a broad range of benefits in sunscreen, paint, coloring, plastics, medicines, papers, toothpaste, inks, cosmetics and food. The daily utility of titanium dioxide is in manufacturing, such as disposable wrappers, which help consumers to save food items from contamination. Titanium dioxide is a white color in a variety of foods, for example, coffee creamer, candy, white sauces and cake decorations. It is an additive in foods in the form of E171 [5], which helps to preserve food for a considerable time period. Recently, various methods and techniques put heads together for the enrichment of the thermal properties of nanofluids [6,7,8,9,10,11,12,13,14,15,16,17]. 

The energy losses due to entropy generation analysis have diverse utilizations in the physical sciences. For example, the characteristics of energy loss for radiative mixed convection flow passing through the vertical channel was reported by Mahmud and Fraser [18]. The effects of magnetic field and the group parameter illustrated subdue behavior on entropy generation, as compared to the mixed convection and radiation parameter. Rashidi et al. [19] have designed the entropy generation of magnetically developing a nanofluid flow for a rotating porous disk. A continuous reduction was noted in the average entropy generation number for the larger value of the nanoparticle volume fraction, while increasing values of magnetic parameter produced an escalation in the average entropy generation number. Numerical analysis for entropy generation on nanofluid with the suspension of nanoparticles, such as copper, Al_2_O_3_ and TiO_3_ in water as a base fluid, which passes through wavy walls, was performed by Cho et al. [20]. They testified that for a given nanofluid, the total energy loss could be minimized and the mean heat transfer number maximized through a suitable adjustment of the wavy surface geometry parameters. Ranjit and Shit [21] carried out the results of entropy generation on electro-osmotic flow with magnetic and Joule heating. They perceived that entropy generation near the channel wall rapidly increased with the increase of the joule heating parameter. A few remarkable contributions on entropy generation with diverse studies can be explored in References [22,23,24,25,26,27,28].

The existing literature firmly endorses that the effective influences of electro-magnetohydrodynamics (EMHD) and entropy generation with nanoparticles through a wavy channel on Poiseuille flow synthesis, are yet not to be addressed. In addition, what made this investigation more fruitful was to observe the simultaneous application of electro-hydrodynamics (EHD) and thermal radiation. The average entropy generation with buoyancy force, yielded a nonlinear coupled relationship. To achieve a formidable and reliable solution of such a nonlinear flow problem, the homotopy analysis method [29] was designated. This method has been used effectively for the last decade [30,31,32]. A brief outline of the succeeding mode of this study is as follows: In the first step, the description and mathematical modeling of flow problem are offered, then the physical quantities, analytical solution, code validation, graphics and numeric results average entropy generation are given. The concluding remarks are briefly listed at the end.

## 2. Problem Description

### 2.1. Flow Analysis

**Figure 1:**
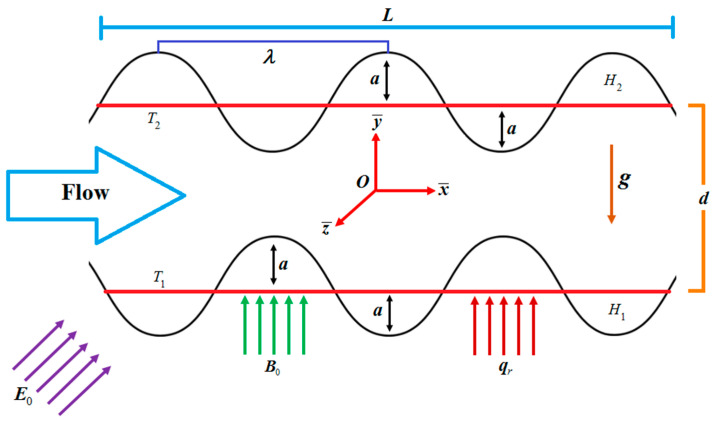
Nanofluid flow model.

An incompressible, electrically conducting, steady-state laminar TiO_2_-water nanofluid flowing between horizontal wavy channels, as displayed in Figure 1, is considered. The lower and upper walls of channel having length L with amplitude a and width d are $H_{1}=-a\mathrm{Cos}\left(2\pi \bar{x}/L\right)-d$ and $H_{2}=a\mathrm{Cos}\left(2\pi \bar{x}/L\right)+d$ respectively.

### 2.2. Formulation

The nanofluid flow model with mixed convection under gravitational force [33,34,35] could be framed as:

(1) ∇.V= 0

(2) $$\left(V.\nabla \right)V=\frac{1}{\rho_{nf}}\left[-\nabla \bar{p}+\mu_{nf}\nabla^{2}V\;+J\times B\right]+\;\beta_{nf}\left(\bar{T}-T_{2}\right)g$$

(3) $${\left(\rho C_{p}\right)}_{nf}\left(V.\nabla \right)\bar{T}=\;k_{nf}\nabla^{2}\bar{T}+\Phi +\frac{1}{\sigma_{nf}}J.J-\nabla .q_{r}$$

Equations (1)–(3) hold: Flow vector V, current density J, gravitational acceleration vector g, dimensional temperature $\bar{T}$, dimensional pressure $\bar{p}$, viscous dissipation Φ, radiative heat flux $q_{r}$, current density J = $\sigma_{nf}\left[\left(V\times B\right)+E\right]$, uniform magnetic field B = $\left[0,B_{0},0\right]$, uniform electric field E = $\left[0,0,-E_{0}\right]$, gravitational acceleration g = [0,g,0], heat flux $q_{r}=\left[0,q_{r},0\right]$, Lorentz force J×B = $\sigma_{nf}\left[E_{0}B_{0}-B_{0}^{2}\bar{u},0,0\right]$ with Joule’s heating J.J = $\sigma_{nf}^{2}{\left(B_{0}\bar{u}-E_{0}\right)}^{2}$ and viscous dissipation $\Phi =\mu_{nf}{\left(\frac{\partial \bar{u}}{\partial \bar{y}}\right)}^{2}$.

Equations (1)–(3) in component form are:

(4) $$\frac{\partial \bar{u}}{\partial \bar{x}}+\frac{\partial \bar{u}}{\partial \bar{y}}=0$$

(5) $$\underset{\mathrm{Inertial}\;\mathrm{term}}{\underset{︸}{\rho_{nf}\left(\bar{u}\frac{\partial \bar{u}}{\partial \bar{x}}+\bar{v}\frac{\partial \bar{u}}{\partial \bar{y}}\right)}}=-\underset{\mathrm{Pressure}\;\mathrm{gradient}}{\underset{︸}{\frac{\partial \bar{p}}{\partial \bar{x}}}}+\underset{\mathrm{viscous}\;\mathrm{term}}{\underset{︸}{\mu_{nf}\left(\frac{\partial^{2}\bar{u}}{\partial {\bar{x}}^{2}}+\frac{\partial^{2}\bar{u}}{\partial {\bar{y}}^{2}}\right)}}+\underset{\mathrm{convection}}{\underset{︸}{{\left(\rho \beta \right)}_{nf}\left(\bar{T}-T_{2}\right)g}}+\underset{\mathrm{External}\;\mathrm{forces}}{\underset{︸}{\sigma_{nf}\left(E_{0}B_{0}-B_{0}^{2}\bar{u}\right)}}$$

(6) $$\underset{\mathrm{Inertial}\;\mathrm{term}}{\underset{︸}{{\left(\rho C_{p}\right)}_{nf}\left(\bar{u}\frac{\partial \bar{T}}{\partial \bar{x}}+\bar{v}\frac{\partial \bar{T}}{\partial \bar{y}}\right)}}=\underset{\mathrm{Heat}\;\mathrm{Conductivity}}{\underset{︸}{k_{nf}\left(\frac{\partial^{2}\bar{T}}{\partial {\bar{x}}^{2}}+\frac{\partial^{2}\bar{T}}{\partial {\bar{y}}^{2}}\right)}}+\underset{\mathrm{Dissipation}}{\underset{︸}{\mu_{nf}{\left(\frac{\partial \bar{u}}{\partial \bar{y}}\right)}^{2}+\sigma_{nf}{\left(B_{0}\bar{u}-E_{0}\right)}^{2}}}-\underset{\mathrm{Radiation}}{\underset{︸}{\frac{\partial q_{r}}{\partial \bar{y}}}}$$

The allied boundary conditions are:

(7) $$\begin{array}{l} \bar{u}=0,\;\bar{v}=0,\;\bar{T}=T_{1}\;\mathrm{at}\;\bar{y}=H_{1}\\ \bar{u}=0,\;\bar{v}=0,\;\bar{T}=T_{2}\;\mathrm{at}\;\bar{y}=H_{2}\;. \end{array}$$

The interrelated viscosity and thermal conductivity models [36] are:

(8) $$\begin{array}{ll} \mu_{nf}=\left(1.0226+0.0477\phi -0.0112\phi^{2}\right)\mu_{f}; & T=15\;{}^{\circ }C\\ \mu_{nf}=\left(1.013+0.092\phi -0.015\phi^{2}\right)\mu_{f}; & T=25\;{}^{\circ }C\\ \mu_{nf}=\left(1.018+0.112\phi -0.0177\phi^{2}\right)\mu_{f}; & T=35\;{}^{\circ }C \end{array}\}$$

(9) $$\begin{array}{l} k_{nf}=\left(1.0225+0.0272\phi \right)k_{f};T=15\;{}^{\circ }C\\ k_{nf}=\left(1.0204+0.0249\phi \right)k_{f};T=25\;{}^{\circ }C\\ k_{nf}=\left(1.0139+0.0250\phi \right)k_{f};T=35\;{}^{\circ }C \end{array}\}$$

The most imperative nanofluid models for density $\rho_{nf}$ [37], heat capacity ${\left(C_{p}\right)}_{nf}$ [38], thermal coefficient $\beta_{nf}$ [39] and electrical conductivity $\sigma_{nf}$ [40] with a nanoparticle volume fraction ϕ are referred for detailed study of the readers.

The Rosseland approximation for radiative heat flux $q_{r}$ [41] is:

(10) $$q_{r}=-\frac{16T_{2}{}^{3}\sigma *}{3k*}\frac{\partial \bar{T}}{\partial \bar{y}}$$

By using the following transformation:

(11) $$\begin{array}{l} x=\frac{\bar{x}}{\lambda },\;u=\frac{\bar{u}}{U_{m}},h_{1}=\frac{H_{1}}{d},\theta =\frac{\bar{T}-T_{2}}{T_{1}-T_{2}},p=\frac{d^{2}\bar{p}}{\mu_{f}U_{m}\lambda },\\ y=\frac{\bar{y}}{d},v=\frac{\bar{v}}{U_{m}\delta },h_{2}=\frac{H_{2}}{d},\delta =\frac{d}{\lambda }. \end{array}$$

Equations (4)–(6) in a dimensionless form is acquired as:

(12) $$\frac{\partial u}{\partial x}+\frac{\partial v}{\partial y}=0,$$

(13) $$A_{2}Re\delta \left(u\frac{\partial u}{\partial x}+v\frac{\partial u}{\partial y}\right)=A_{1}\left[\left(\delta^{2}\frac{\partial^{2}u}{\partial x^{2}}+\frac{\partial^{2}u}{\partial y^{2}}\right)-\frac{\partial p}{\partial x}\right]+A_{3}M^{2}\left(E_{1}-u\right)+A_{4}Gr\theta ,$$

(14) $$A_{5}RePr\delta \left(u\frac{\partial \theta }{\partial x}+v\frac{\partial \theta }{\partial y}\right)=A_{6}\left(\delta^{2}\frac{\partial^{2}\theta }{\partial x^{2}}+\frac{\partial^{2}\theta }{\partial y^{2}}\right)+A_{1}EcPr{\left(\frac{\partial u}{\partial y}\right)}^{2}+A_{3}EcPrM^{2}{\left(u-E_{1}\right)}^{2}+Rd\left(\frac{\partial^{2}\theta }{\partial y^{2}}\right).$$

Where:

(15) $$\begin{array}{l} Gr=\frac{{\left(\rho \beta \right)}_{f}gd^{2}\left(T_{1}-T_{2}\right)}{\mu_{f}U_{m}},Re=\frac{\rho_{f}U_{m}d}{\mu_{f}},M=\frac{\sigma_{f}B_{0}{}^{2}d^{2}}{\mu_{f}},E_{1}=\frac{E_{0}}{B_{0}U_{m}}\\ Pr=\frac{\mu_{f}{\left(\rho C_{p}\right)}_{f}}{\rho_{f}k_{f}},Ec=\frac{U_{m}^{2}}{{\left(C_{p}\right)}_{f}\left(T_{1}-T_{2}\right)},Br=PrEc,Rd=\frac{16T_{2}{}^{3}\sigma *}{3k*k_{f}}\\ A_{1}=\frac{\mu_{nf}}{\mu_{f}},\;A_{2}=\frac{\rho_{nf}}{\rho_{f}},\;A_{3}=\frac{\sigma_{nf}}{\sigma_{f}},A_{4}=\frac{{\left(\rho \beta \right)}_{nf}}{{\left(\rho \beta \right)}_{f}},A_{5}=\frac{{\left(\rho C_{p}\right)}_{nf}}{{\left(\rho C_{p}\right)}_{f}},\;A_{6}=\frac{k_{nf}}{k_{f}}. \end{array}\}$$

The key parameters contain wavelength λ, non-dimensional wave number δ, non-dimensional velocity components (u,v), lower wall temperature $T_{1}$, upper wall temperature $T_{2}$ and dimensionless temperature θ. When a fluid is moving with a constant pressure gradient with: $U_{m}=-\frac{a^{2}}{2\mu_{f}}\frac{\partial p}{\partial x}$ then $\frac{\partial p}{\partial x}=P$ as given by Reference [42].

Under the long wavelength approximation, Equations (12)–(14) along with linked boundary in a dimensionless form are renewed as:

(16) $$-A_{1}P+A_{1}\frac{\partial^{2}u}{\partial y^{2}}+A_{3}M\left(E_{1}-u\right)+A_{4}Gr\theta =0$$

(17) $$\left(A_{6}+Rd\right)\frac{\partial^{2}\theta }{\partial y^{2}}+A_{1}EcPr{\left(\frac{\partial u}{\partial y}\right)}^{2}+A_{3}EcPrM{\left(u-E_{1}\right)}^{2}=0$$

(18) $$\begin{array}{l} u=0,\theta =1\;\mathrm{at}\;y=h_{1}\;\mathrm{where}\;h_{1}=-1-\frac{a\mathrm{Cos}\left(2\pi x\lambda /L\right)}{d}\\ u=0,\theta \;=\;0\;\mathrm{at}\;y=h_{2}\;\mathrm{where}\;h_{2}=1+\frac{a\mathrm{Cos}\left(2\pi x\lambda /L\right)}{d} \end{array}\}\;$$

The significant characteristics of nanoparticles with a base fluid are specified in Table 1.

### 2.3. Physical Quantities

#### 2.3.1. Drag Force (Skin Friction)

The drag force $C_{f}$ [45] is well-defined by:

(19) $$C_{f}=\frac{2\tau_{w}}{\rho_{f}U_{m}{}^{2}},\;\mathrm{with}\;\tau_{w}(\mathrm{wall}\;\mathrm{shear}\;\mathrm{stress})\;={\mu_{nf}\left(\frac{\partial \bar{u}}{\partial \bar{y}}\right)|}_{\bar{y}=H_{1}\;and\;H_{2}}$$

By using Equations (11) and (15), and neglecting dimensionless flow properties, Equation (19) is reformed as:

(20) Cf=2A1Reu′(y)|y=h1 and h2

#### 2.3.2. Heat Transfer Ratio (Nusselt Number)

The Heat transfer ratio Nu, [46] may be laid out as:

(21) $$Nu=\frac{dq_{w}}{k_{f}\left(T_{1}-T_{2}\right)},\;\mathrm{with}\;q_{w}(\mathrm{wall}\;\mathrm{heat}\;\mathrm{fl}\mathrm{ux})\;=\;{k_{nf}\left(\frac{\partial \bar{T}}{\partial \bar{y}}\right)|}_{\bar{y}=H_{1}\;and\;H_{2}}$$

With the same analogy, in view of Equations (11) and (15), by neglecting the flow properties in a dimensionless form of Equation (21), one has:

(22) $$Nu=-{A_{6}\theta^{\prime }\left(y\right)|}_{y=h_{1}\;\mathrm{and}\;h_{2}}$$

## 3. Analysis of Energy Loss

The local entropy generation $E_{G}$ in a nanofluid with effective influences of electro-magnetohydrodynamics (EMHD) and thermal radiative heat flux is described in the subsequent relation as:

(23) $$E_{G}=\underset{\begin{array}{l} \mathrm{energy}\;\mathrm{loss}\;\mathrm{via}\\ \mathrm{heat}\;\mathrm{transfer} \end{array}}{\underset{︸}{\frac{k_{nf}}{T_{2}{}^{2}}\left[{\left(\frac{\partial \bar{T}}{\partial \bar{y}}\right)}^{2}-q_{r}\left(\frac{\partial \bar{T}}{\partial \bar{y}}\right)\right]}}+\underset{\begin{array}{l} \mathrm{energy}\;\mathrm{loss}\;\mathrm{via}\\ \;\mathrm{fluid}\;\mathrm{friction} \end{array}}{\underset{︸}{\frac{\mu_{nf}}{T_{2}}{\left(\frac{\partial \bar{u}}{\partial \bar{y}}\right)}^{2}}}+\underset{\begin{array}{l} \mathrm{energy}\;\mathrm{loss}\;\mathrm{via}\\ \mathrm{Joule}\;\mathrm{dissipation}\\ \mathrm{and}\;\mathrm{electric}\;\mathrm{field} \end{array}}{\underset{︸}{\frac{\sigma_{nf}{\left(B_{0}\bar{u}-E_{0}\right)}^{2}}{T_{2}}}}$$

The entropy generation rate $E_{G_{0}}$ is determined by:

(24) $$E_{G_{0}}=\frac{k_{nf}{\left(T_{1}-T_{2}\right)}^{2}}{d^{2}T_{2}{}^{2}}$$

The entropy generation number $N_{G}$ is signified in b.

(25) $$N_{G}=E_{G}/E_{G_{0}}$$

Such that:

(26) $$N_{G}=\frac{d^{2}T_{2}{}^{2}}{k_{nf}{\left(T_{1}-T_{2}\right)}^{2}}\times \left[\frac{k_{nf}}{T_{2}{}^{2}}\left[{\left(\frac{\partial \bar{T}}{\partial \bar{y}}\right)}^{2}-q_{r}\left(\frac{\partial \bar{T}}{\partial \bar{y}}\right)\right]+\frac{\mu_{nf}}{T_{2}}{\left(\frac{\partial \bar{u}}{\partial \bar{y}}\right)}^{2}+\frac{1}{T_{2}}\sigma_{nf}{\left(B_{0}\bar{u}-E_{0}\right)}^{2}\right]$$

Hence, the total entropy generation is:

(27) $$N_{G}=\left(1+\frac{4}{3}Rd\right){\left(\frac{\partial \theta }{\partial y}\right)}^{2}+\frac{1}{A_{6}}\frac{Br}{\Omega }\left[A_{1}{\left(\frac{\partial u}{\partial y}\right)}^{2}+A_{3}M^{2}{\left(u-E_{1}\right)}^{2}\right]$$

Where:

(28) $$\Omega =\frac{T_{1}-T_{2}}{T_{2}},Br=\frac{\mu_{f}U_{m}^{2}}{k_{f}\left(T_{1}-T_{2}\right)}$$

The Bejan number Be can be made as:

(29) $$Be=\frac{HTI}{HTI+FFI+JDEI}$$

(30) $$HTI={\left(\frac{\partial \theta }{\partial y}\right)}^{2},FFI=\frac{A_{1}}{A_{6}}\frac{Br}{\Omega }{\left(\frac{\partial u}{\partial y}\right)}^{2},JDEI=\frac{A_{3}}{A_{6}}\frac{Br}{\Omega }M^{2}{\left(u-E_{1}\right)}^{2}$$

From Equations (29) and (30), it follows that:

(31) $$Be=\frac{\left(1+\frac{4}{3}Rd\right){\left(\frac{\partial \theta }{\partial y}\right)}^{2}}{\left(1+\frac{4}{3}Rd\right){\left(\frac{\partial \theta }{\partial y}\right)}^{2}+\frac{1}{A_{6}}\frac{Br}{\Omega }\left[A_{1}{\left(\frac{\partial u}{\partial y}\right)}^{2}+A_{3}M^{2}{\left(u-E_{1}\right)}^{2}\right]}$$

Average entropy generation is calculated through:

(32) $$N_{G\_avg}=\frac{1}{\forall }\int_{\forall }N_{G}\;d\forall$$

Here:

(33) $$N_{G\_avg}=\frac{1}{\left(d\times L\right)}\int_{h_{1}}^{h_{2}}N_{G}\;dy$$

Or:

(34) $$,N_{G\_avg}=\frac{1}{\left(d\times L\right)}\int_{h_{1}}^{h_{2}}\left(HTI+FFI+JDEI\right)\;dy$$

## 4. Analytical Procedure

The initial guesses $u_{0}\left(y\right)$, $\theta_{0}\left(y\right)$ along with the linear operators ${£}_{u}$, ${£}_{\theta }$ are selected as per the criterion given in Reference [47]:

(35) $$u_{0}\left(y\right)=\;y^{2}-(h_{1}+h_{2})y+(h_{1}h_{2});\theta_{0}\left(y\right)=\frac{y-h_{2}}{h_{1}-h_{2}}$$

(36) $${£}_{u}=\frac{d^{2}u}{dy^{2}},{£}_{\theta }=\frac{d^{2}\theta }{dy^{2}}$$

The zeroth-order initial guesses along with the nonlinear operators $N_{u}$, $N_{\theta }$ with the embedding factor ξ∈[0, 1] under the convergence control factors $\hbar_{u}$, $\hbar_{\theta }$ are respectively attained as:

(37) $$\begin{array}{l} \left(1-\xi \right){£}_{u}\left[u\left(y,\xi \right)-u_{0}\left(y\right)\right]-\xi \hbar_{u}N_{u}\left[u\left(y,\;\xi \right),\;\;\theta \left(y,\;\xi \right)\right]=0,\\ \left(1-\xi \right){£}_{\theta }\left[\theta \left(y,\xi \right)-\theta_{0}\left(y\right)\right]-\xi \hbar_{\theta }N_{\theta }\left[u\left(y,\;\xi \right),\;\;\theta \left(y,\;\xi \right)\right]=0. \end{array}\}$$

(38) $$\begin{array}{c} N_{u}\left[u\left(y,\;\xi \right),\;\theta \left(y,\;\xi \right)\right]=-A_{1}P+A_{1}\frac{\partial^{2}u\left(y,\;\xi \right)}{\partial y^{2}}+A_{3}M^{2}\left[E_{1}-u\left(y,\;\xi \right)\right]+A_{4}Gr\theta \left(y,\;\xi \right)\\ N_{\theta }\left[u\left(y,\;\xi \right),\;\theta \left(y,\;\xi \right)\right]=\left(A_{6}+Rd\right)\frac{\partial^{2}\theta \left(y,\;\xi \right)}{\partial y^{2}}+A_{1}EcPr{\left(\frac{\partial u\left(y,\;\xi \right)}{\partial y}\right)}^{2}+\\ A_{3}EcPrM^{2}{\left(u\left(y,\;\xi \right)-E_{1}\right)}^{2} \end{array}\}\;$$

(39) $$\mathrm{for}\;\xi =0\text{:}\;u\left(y,\xi \right)=u_{0}\left(y\right)\;\mathrm{and}\;\theta \left(y,\;\xi \right)=\theta_{0}\left(y\right)$$

(40) for ξ=1: u(y,ξ)=u(y) and θ(y, ξ)=θ(y)

Tiresome mathematical manipulation leads to analytical expressions of velocity and thermal energy respectively, by the following equations:

(41) $$\begin{array}{r} u(y)=u_{0}\left(y\right)\;+\sum_{k=1}^{l}u_{k}\left(y\right)\\ \;\theta (y)=\theta_{0}\left(y\right)\;+\sum_{k=1}^{l}\theta_{k}\left(y\right)\; \end{array}\}$$

## 5. Code Validation and Convergence Analysis

**Figure 2:**
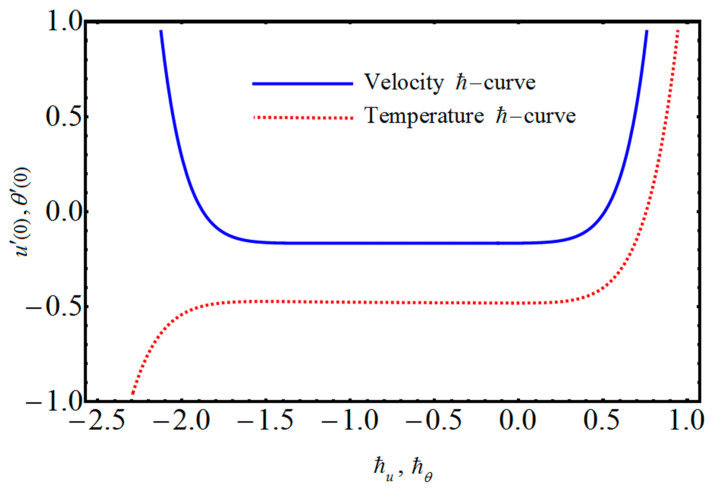
ℏ-curves.

The velocity and temperature results in Equation (41) contain the auxiliary parameters $\hbar_{u}$ and $\hbar_{\theta }$, respectively. As pointed out by the originator of homotopy analysis method, a faster convergence can be achieved by the optimum selection of the involved auxiliary parameters [48]. Figure 2 portrays the ℏ-curves at thirtieth-order approximations for velocity and temperature, to estimate the suitable interval of convergence, that visibly predicts admissible ranges for $\hbar_{u}$ and $\hbar_{\theta }$ to lie between −2.0 to 0.5 and −1.5 to 0.5.

For the optimum values of $\hbar_{u}$ and $\hbar_{\theta }$, the residual errors were computed up to thirtieth-order approximations over an embedding parameter ξ∈[0, 1] of velocity $E_{u}$ and temperature distributions $E_{\theta }$, by the succeeding formulas:

(42) $$E_{u}=\sqrt{\frac{1}{31}\sum_{i=0}^{30}{\left(u\left(i/30\right)\right)}^{2}}\;\mathrm{and}\;E_{\theta }=\sqrt{\frac{1}{31}\sum_{j=0}^{30}{\left(\theta \left(j/30\right)\right)}^{2}}$$

**Figure 3:**
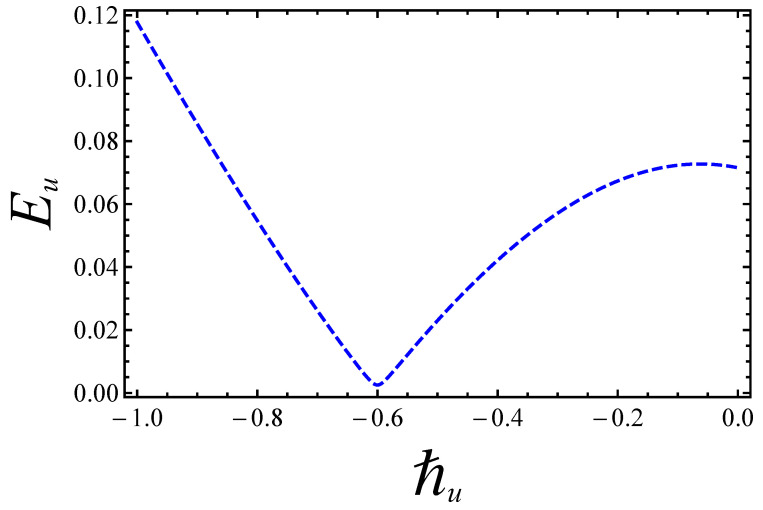
Residual error for velocity profile.

**Figure 4:**
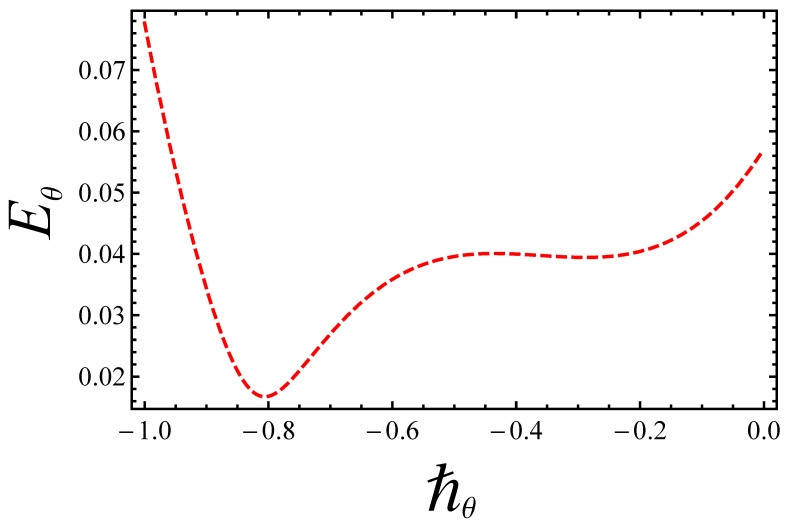
Residual error for temperature profile.

Eventually, Figure 3 and Figure 4 bear witness that the best optimum values of the ℏ-curves for velocity and temperature, within admissible ranges, are $\hbar_{u}=-0.6$ and $\hbar_{\theta }=-0.8$, respectively. The residual errors for the convergence of analytical solutions are further elaborated in Table 2.

## 6. Results and Discussion

**Figure 5:**
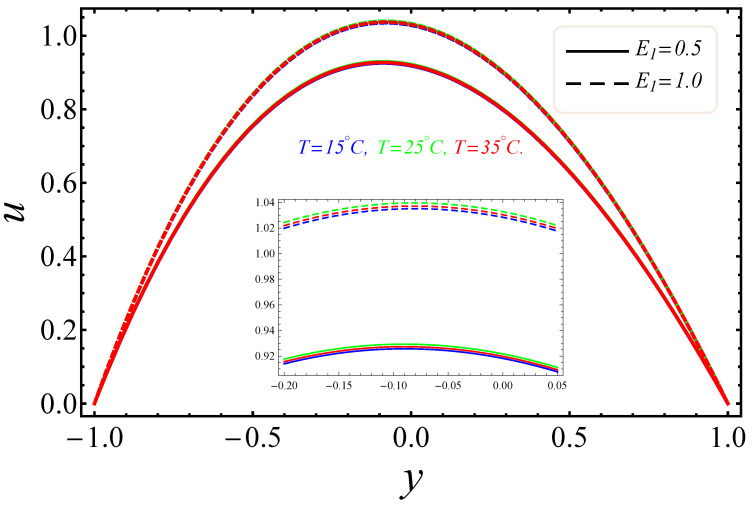
Manifestation of $E_{1}$ on u.

**Figure 6:**
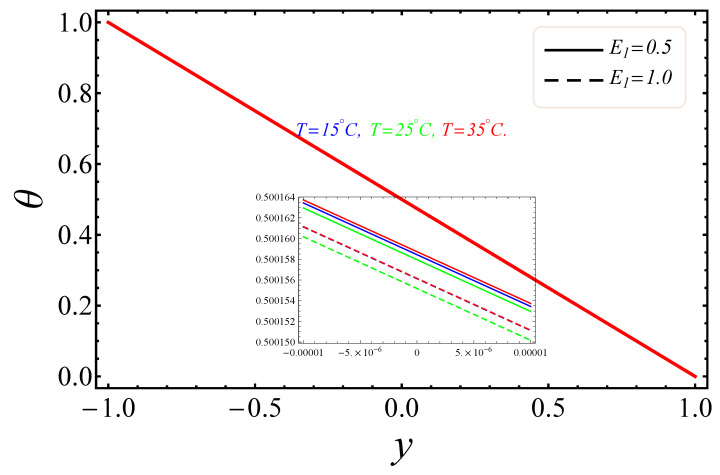
Manifestation of $Br\Omega^{-1}$ on θ.

**Figure 7:**
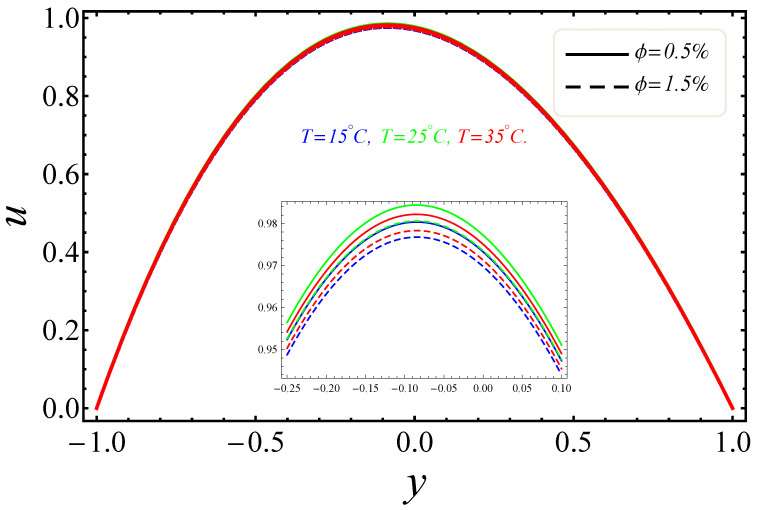
Manifestation of ϕ on u.

**Figure 8:**
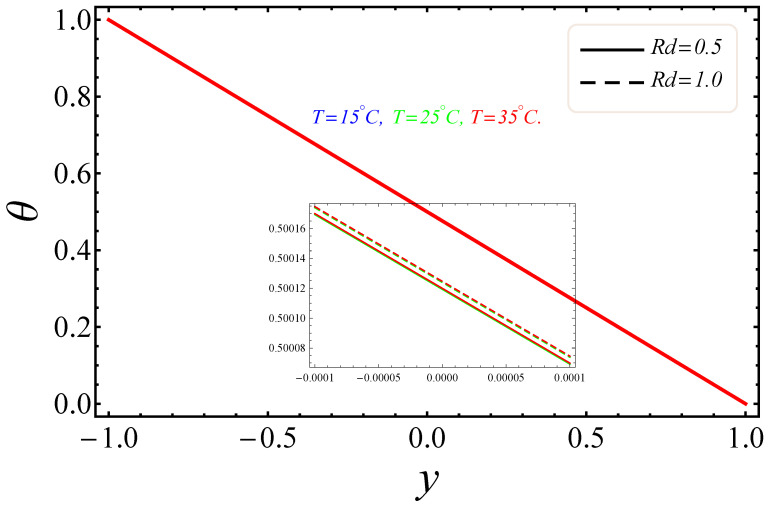
Manifestation of Rd on θ.

The sketches of the key factors, such as the electric field, nanoparticle volume fraction, radiation and group parameter are presented for three different temperatures (15 °C, 25 °C, 35 °C). Figure 5, Figure 6, Figure 7 and Figure 8 signify the impressions of $E_{1}$, ϕ and Rd on the velocity (u) and temperature (θ) profiles. The plots of the electric field parameter $E_{1}$ on the velocity and temperature distributions are shown in Figure 5 and Figure 6. Figure 5, identified that the velocity gradually increased by an upturn of $E_{1}$, whereas the combined effects of the electro-magnetohydrodynamics (EMHD) produced Lorentz forces to resist the fluid velocity. Also, the thickness of the boundary layer increased with the rise of $E_{1}$. However, in Figure 6, the opposite behavior for the fluid temperature was noted, which was due to the applied electric field. The effects of the nanoparticle volume fraction ϕ on the fluid flow are shown in Figure 7. It could easily be examined that when the volume fraction of the nanoparticle upsurges in the base fluid, the base fluid’s density increased. Subsequently, the fluid became denser, so the suspension of the particles in the fluid resulted in a reduction in nanofluid velocity. In Figure 8, the temperature distribution of the nanofluid against the radiation parameter Rd is displayed. The boundary layer thickness increased with increasing values of the radiation factor. The temperature of nanofluid could also be controlled with the radiation factor, because the fluid temperature was very sensitive to Rd, which meant that the heat flux of channel walls would be as large as perceived.

**Figure 9:**
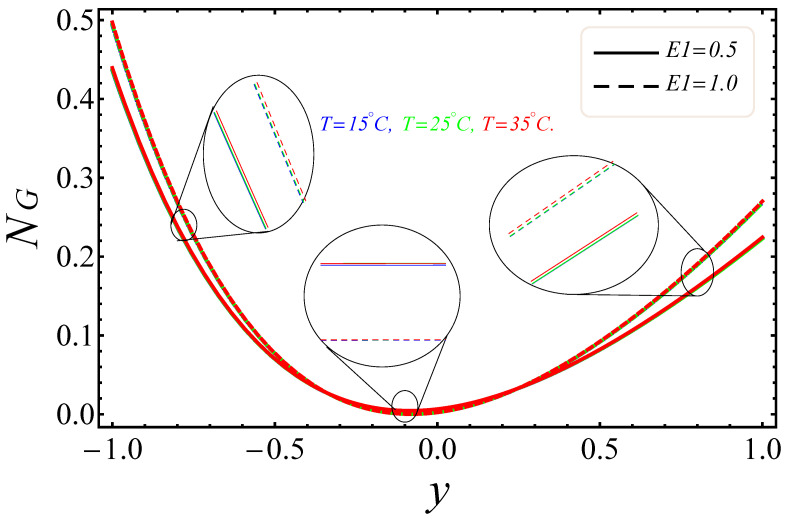
Manifestation of $E_{1}$ on $N_{G}$.

**Figure 10:**
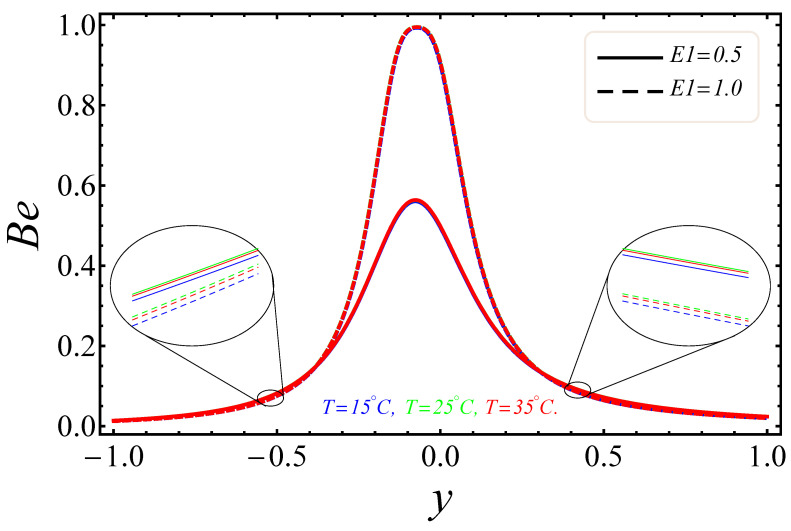
Manifestation of $E_{1}$ on Be.

**Figure 11:**
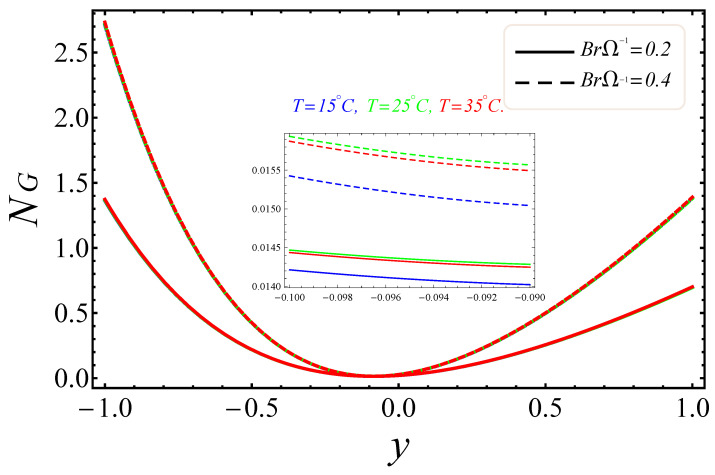
Manifestation of $Br\Omega^{-1}$ on $N_{G}$.

**Figure 12:**
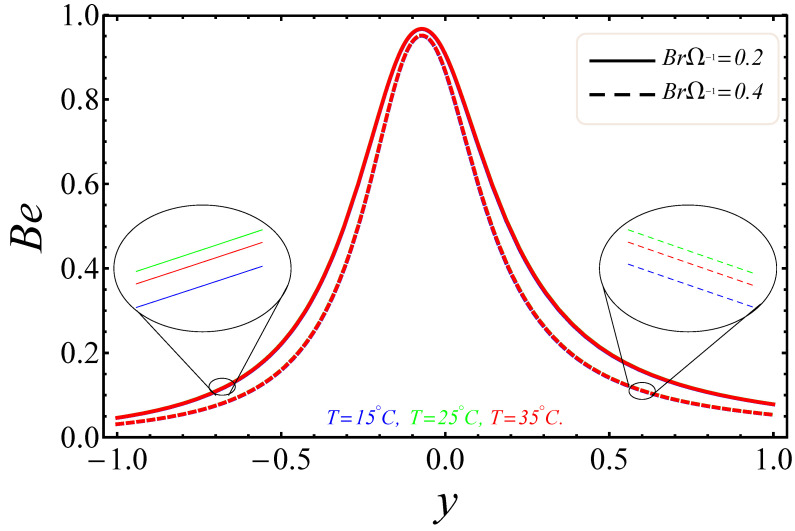
Manifestation of $Br\Omega^{-1}$ on Be.

**Figure 13:**
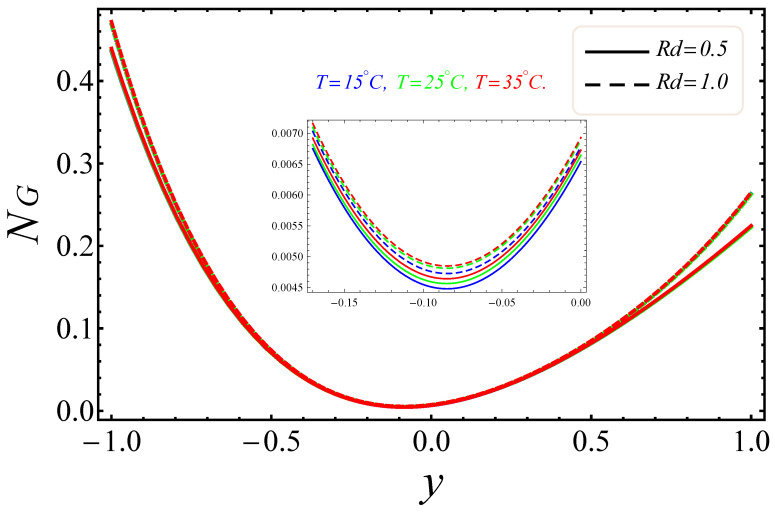
Manifestation of Rd on $N_{G}$.

**Figure 14:**
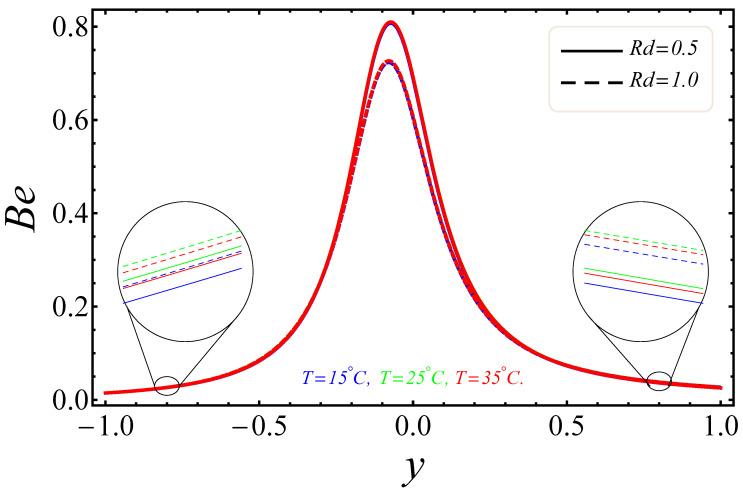
Manifestation of Rd on Be.

Figure 9, Figure 10, Figure 11, Figure 12, Figure 13 and Figure 14 portray the effects of $E_{1}$, Br/Ω and Rd on $N_{G}$ and Be. Figure 9 and Figure 10 show the behaviors of the electric field parameter $E_{1}$ on $N_{G}$ and Be. The entropy generation rate near the walls increased with the increase of the electric field parameter, as shown in Figure 9, while at the left wall, the entropy loss was greater as compared to the right wall. It is further noted that near the center of the channel, energy loss was at a minimum, between y=−0.3 and y=0.2. This was due to the combined effects of the electro-magnetohydrodynamics, which produced Lorentz forces to resist the fluid flow. In Figure 10, The Bejan number near to the center of the channel with a large electric parameter value gradually accelerated and approached to 1, but near to the walls, a reduction in the Bejan number against large values of the electric field parameter was detected. The impacts of group parameters Br/Ω on $N_{G}$ and Be are shown in Figure 11 and Figure 12. The entropy generation rate escalated with increasing values of the group parameter, as shown in Figure 11. One also noticed that the entropy generation rate at the left wall as compared to the right wall was high due to the increase in buoyancy forces in the system. The upshot of Br/Ω was visible in Figure 12. Here Be attained an extreme value, almost at y=−0.1, because of the escalation of the heat transfer irreversibility for Br/Ω=0.2, but gradually decreased with the increase of the group parameter values. The effects of the radiation parameter Rd on the entropy generation rate are displayed in Figure 13. Here, the entropy generation was characterized by the nice concave shape and almost symmetrical profiles for all values of Rd. A small change in Rd caused a large variation of $N_{G}$, as seen in Figure 13. It could also be noted that the energy loss entropy generation rate round the center of the channel was approximately zero, but as one proceeded towards the channel walls, entropy occurred. Figure 14 shows the same increasing results for the radiation parameter Rd on the Bejan number Be, as shown in the case of entropy generation. The Bejan number near the center of the channel was about to attain its extreme position for low radiation evolvement, but near the vicinity of the walls, the Bejan number increased with the growing radiation factor. The increasing results suggested that heat transfer irreversibility plays a dominant role in energy loss.

**Figure 15:**
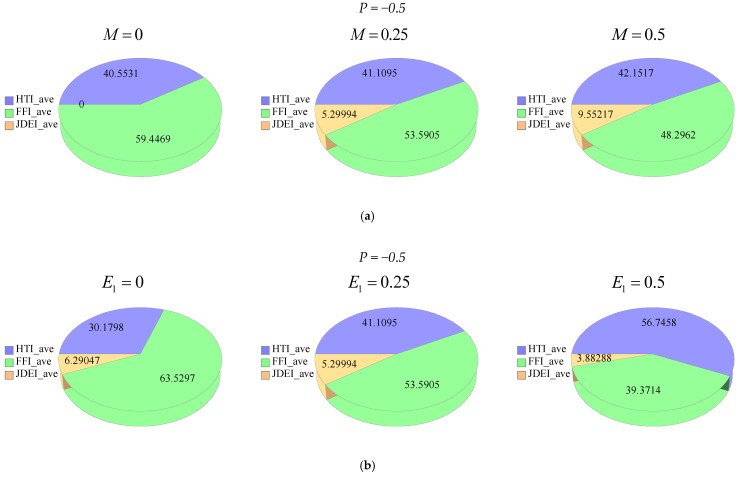

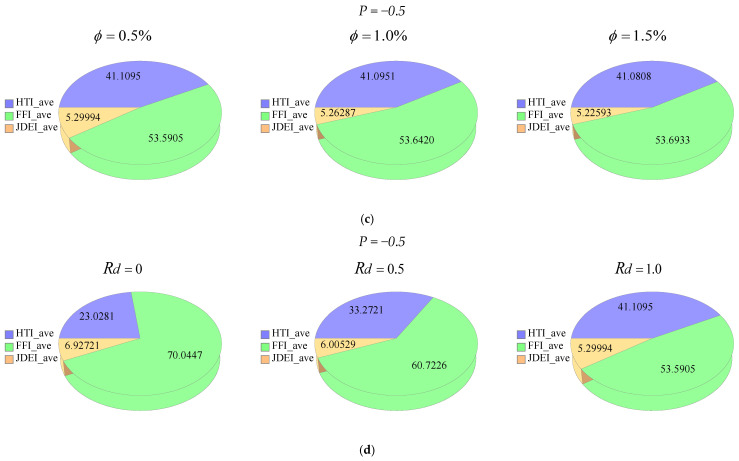
(**a**) Phi diagrams showing effects of magnetic parameter by keeping other parameters as fixed. (**b**) Phi diagrams showing effects of electric field parameter by keeping other parameters as fixed. (**c**) Phi diagrams showing effects of nanoparticles volume fraction by keeping other parameters as fixed. (**d**) Phi diagrams showing effects of radiation parameter by keeping other parameters as fixed.

**Figure 16:**
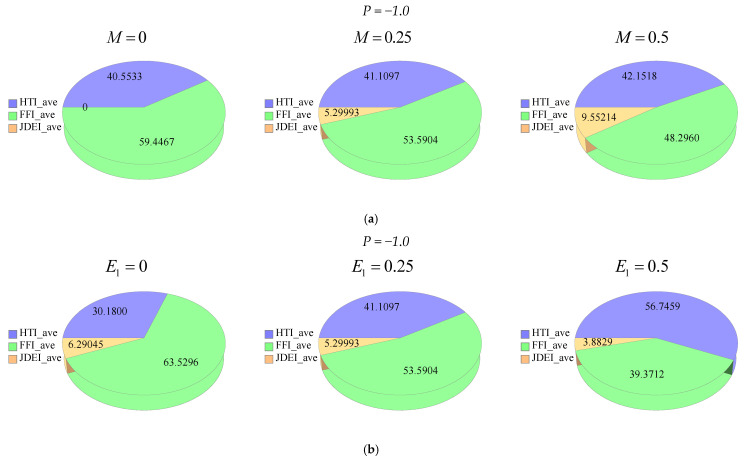

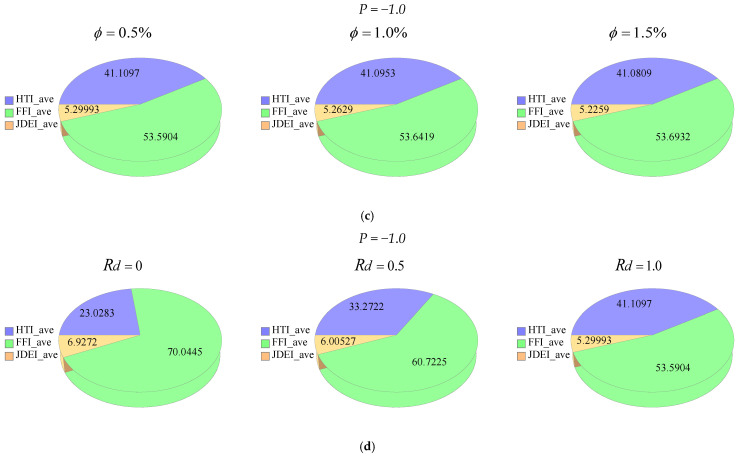
(**a**) Phi diagrams showing effects of magnetic parameter by keeping other parameters as fixed. (**b**) Phi diagrams showing effects of electric field parameter by keeping other parameters as fixed. (**c**) Phi diagrams showing effects of nanoparticles volume fraction by keeping other parameters as fixed. (**d**) Phi diagrams showing effects of radiation parameter by keeping other parameters as fixed.

Figure 15a–d and Figure 16a–d depict the effects of M, $E_{1}$, ϕ and Rd on: The average heat transfer irreversibility (HTI average), average fluid friction irreversibility (FFI average), average joule dissipation and electric field irreversibility (JDEI average) by using Duangthongsuk and Wongwises’ [36] model at $T=25\;{}^{\circ }$C. In Figure 15a, phi diagrams are displayed against the magnetic parameter for different M. In Figure 15b, the phi diagrams show the performance of the electric field for different $E_{1}$. In Figure 15c, the phi drawings deal with the nanoparticle volume fraction for different ϕ. In Figure 15d, the phi drawings describe the radiation parameter for different values of Rd. The effects of the magnetic parameter for different values of M are given in the phi diagrams, as shown in Figure 16a, whereas Figure 16b, show the phi diagrams against the electric field parameter for different values of $E_{1}$. In Figure 16c, the phi diagrams depict the effects of the nanoparticle volume fraction for different values of ϕ, while Figure 16d, show the effects of the radiation parameter for different values of Rd via phi diagrams. In all phi diagrams, it was determined that when the pressure gradient increased, the average entropy loss and consequently entropy generation increased in the system. Thus, one can say that the reported results about electro-magnetohydrodynamics (EMHD), thermal radiation and entropy generation on Poiseuille flow with Titanium dioxide nanoparticles are very effective to reduce the energy losses and escalate the heat transfer in wavy surfaces. The said analysis is very informative for food industries, as in the presence of titanium dioxide in the consumer packaging, which helps to preserve food for a considerable time period. 

The numeric features of $C_{f}$ and Nu on both opposite walls—with respect to three different temperature/conditions, as suggested by Duangthongsuk and Wongwises [36]—against different values of the nanoparticle, volume fraction, electric element and magnetic factor, are calculated in Table 3 and Table 4, respectively. It could be noted that the skin friction reduced at the lower wall, with increasing values of ϕ, $E_{1}$ and M, while the opposite effects occurred at the wall of the concerned parameters. In heat transfer phenomena, the heat rate increased at the lower wall but decreased at the upper wall, with large values of ϕ, $E_{1}$ and M.

## 7. Conclusions

The electro-magnetohydrodynamics (EMHD) and entropy generation on the Poiseuille flow synthesis with nanoparticles through a wavy channel were investigated here. The most vital findings were:

1) The electric field $E_{1}$ applied on a tangential direction to the fluid affected both the velocity and temperature distributions, which produced a reduction in the temperature and an increase in the velocity.
2) The suspension of nanoparticles ϕ in the base fluid caused a slowdown in nanofluid velocity.
3) The thermal boundary layer increased against the growing radiation parameter Rd, which was why an increase in temperature was observed.
4) The entropy generation near the boundary of the channel prolonged, while was very insufficient at the vicinity of the center for the electric field $E_{1}$.
5) Initially, Be attained a high impact near the middle of channel, but gradually it fell for a large value of the electric field parameter near the walls.
6) The entropy generation for the group parameter $Br\Omega^{-1}$ and the radiation parameter Rd at the intermediate of the channel was approximately zero, while an enhancement was noted near the walls.
7) The average energy loss was due to a rise in the pressure gradient.

## Figures and Tables

**Table 1:** Characteristics of nanoparticles and base fluid.

Property	Water (H_2_O)	Titanium dioxide (TiO_2_) [43,44]
Density (kgm^−3^)	$\rho_{f}$**=** 9.877 × 10^2^	$\rho_{p}$ **=** 4.250 × 10^3^
Heat capacity (Jkg^−1^ K^−1^)	${\left(C_{p}\right)}_{f}$ **=** 4.066 × 10^3^	${\left(C_{p}\right)}_{p}$ = 6.862 × 10^2^
Electrical conductivity (m^−1^)	$\sigma_{f}$ **=** 5.0 × 10^−2^	$\sigma_{p}$ = 0.24 × 10^7^
Thermal conductivity (W m^−1^ K^−1^)	$k_{f}$ **=** 6.44 × 10^−1^	$k_{p}$ = 8.9538
Thermal expansion coefficient (K^−1^)	$\beta_{f}$ **=** 21 × 10^5^	$\beta_{p}$ = 0.9 × 10^5^

**Table 2:** Residual error estimation when M=0.25, $E_{1}=1.0,$ Gr=2.0, Rd=0.5, and Pr=7.0.

Order of Approximation	Time	$E_{u}$	$E_{\theta }$
05	5.3818	4..4073 × 10^−4^	2.8357 × 10^−6^
10	9.7290	2.8199 × 10^−8^	4.6835 × 10^−9^
15	16.7899	4.0554 × 10^−14^	5.0418 × 10^−14^
20	26.9812	1.0687 × 10^−17^	1..0454 × 10^−17^
30	40.6344	1.3593 × 10^−22^	7.9903 × 10^−22^

**Table 3:** Numeric attributes of $C_{f}$ on opposite walls with respect to three different temperature/conditions against different points of ϕ, $E_{1}$ and M when Gr=2.0 and Rd=1.0.

ϕ	$E_{1}$	M	$T=15\;{}^{\circ }C$		$T=25\;{}^{\circ }C$		$T=35\;{}^{\circ }C$	
			$C_{f}\left(-1\right)$	$C_{f}\left(1\right)$	$C_{f}\left(-1\right)$	$C_{f}\left(1\right)$	$C_{f}\left(-1\right)$	$C_{f}\left(1\right)$
0.5%	0.0	0.00	3.80765	−2.41647	3.79748	−2.40690	3.80302	−2.41211
		0.25	3.56943	−2.19941	3.55835	−2.18901	3.56439	−2.19466
		0.50	3.36696	−2.01740	3.35512	−2.00632	3.36157	−2.01235
	0.5	0.00	3.80765	−2.41647	3.79748	−2.40690	3.80302	−2.41211
		0.25	3.79915	−2.42912	3.78789	−2.41854	3.79402	−2.42430
		0.50	3.79582	−2.44626	3.78342	−2.43462	3.79017	−2.44096
	1.0	0.00	3.80765	−2.41647	3.79748	−2.40690	3.80302	−2.41211
		0.25	4.02891	−2.65888	4.01746	−2.64812	4.02370	−2.65398
		0.50	4.22474	−2.87519	4.21177	−2.86298	4.21883	−2.86962
1.0%	0.0	0.00	3.79649	−2.41100	3.78657	−2.40167	3.79221	−2.40697
		0.25	3.56060	−2.19602	3.54979	−2.18588	3.55594	−2.19164
		0.50	3.35987	−2.01553	3.34833	−2.00473	3.35489	−2.01086
	0.5	0.00	3.79649	−2.41100	3.78657	−2.40167	3.79221	−2.40697
		0.25	3.78872	−2.42414	3.77774	−2.41382	3.78398	−2.41968
		0.50	3.78594	−2.44161	3.77386	−2.43026	3.78073	−2.43671
	1.0	0.00	3.79649	−2.41100	3.78657	−2.40167	3.79221	−2.40697
		0.25	4.01688	−2.65231	4.00572	−2.64181	4.01207	−2.64777
		0.50	4.21208	−2.86774	4.19945	−2.85586	4.20663	−2.86261
1.5%	0.0	0.00	3.78532	−2.40553	3.77565	−2.39643	3.78140	−2.40183
		0.25	3.55174	−2.19262	3.54122	−2.18273	3.54747	−2.18860
		0.50	3.35275	−2.01363	3.34151	−2.00311	3.34818	−2.00935
	0.5	0.00	3.78532	−2.40553	3.77565	−2.39643	3.78140	−2.40183
		0.25	3.77827	−2.41915	3.76758	−2.40909	3.77393	−2.41506
		0.50	3.77605	−2.43693	3.76429	−2.42589	3.77127	−2.43244
	1.0	0.00	3.78532	−2.40553	3.77565	−2.39643	3.78140	−2.40183
		0.25	4.00485	−2.64573	3.99398	−2.63550	4.00044	−2.64157
		0.50	4.19941	−2.86030	4.18712	−2.84873	4.19442	−2.85559

**Table 4:** Numeric attributes of Nu on opposite walls with respect to three different temperature/conditions against different points of ϕ *,* $E_{1}$ and M when Gr=2.0 and Rd=1.0.

ϕ	$E_{1}$	M	$T=15\;{}^{\circ }C$		$T=25\;{}^{\circ }C$		$T=35\;{}^{\circ }C$	
			Nu(−1)	Nu(1)	Nu(−1)	Nu(1)	Nu(−1)	Nu(1)
0.5%	0.0	0.00	0.510991	0.511551	0.509936	0.510495	0.506687	0.507244
		0.25	0.511011	0.511534	0.509955	0.510479	0.506707	0.507228
		0.50	0.511032	0.511517	0.509976	0.510461	0.506728	0.507211
	0.5	0.00	0.510991	0.511551	0.509936	0.510495	0.506687	0.507244
		0.25	0.510989	0.511553	0.509934	0.510497	0.506685	0.507247
		0.50	0.510987	0.511555	0.509932	0.510499	0.506683	0.507249
	1.0	0.00	0.510991	0.511551	0.509936	0.510495	0.506687	0.507244
		0.25	0.510962	0.511613	0.509872	0.510556	0.506623	0.507306
		0.50	0.510863	0.511672	0.509809	0.510616	0.506560	0.507365
1.0%	0.0	0.00	0.511061	0.511618	0.510000	0.510556	0.506752	0.507306
		0.25	0.511081	0.511601	0.510019	0.510540	0.506771	0.507289
		0.50	0.511101	0.3511584	0.510040	0.510523	0.506791	0.507272
	0.5	0.00	0.511061	0.511618	0.510000	0.510556	0.506752	0.507306
		0.25	0.511059	0.511620	0.509998	0.510558	0.506749	0.507308
		0.50	0.511057	0.511622	0.509996	0.510560	0.506747	0.507310
	1.0	0.00	0.511061	0.511618	0.510000	0.510556	0.506750	0.507306
		0.25	0.510997	0.511679	0.509936	0.510617	0.506688	0.507367
		0.50	0.510934	0.511738	0.509873	0.510676	0.506625	0.507425
1.5%	0.0	0.00	0.511131	0.511685	0.510000	0.510617	0.506816	0.507367
		0.25	0.511150	0.511668	0.510083	0.510601	0.506835	0.507351
		0.50	0.511171	0.511651	0.510103	0.510584	0.506855	0.507334
	0.5	0.00	0.511131	0.511685	0.510064	0.510617	0.506816	0.507367
		0.25	0.511129	0.511687	0.510062	0.510619	0.506814	0.507369
		0.50	0.511126	0.511689	0.510060	0.510621	0.5068110	0.507371
	1.0	0.00	0.511131	0.511685	0.510064	0.510617	0.506816	0.507428
		0.25	0.511067	0.511746	0.510000	0.510678	0.506752	0.507428
		0.50	0.511004	0.511804	0.509938	0.510736	0.506690	0.507486
